# CASE REPORT An Unusual Case of Abdominal Compartment Syndrome Following Resection of Extensive Posttraumatic Mesenteric Ossification

**Published:** 2013-03-07

**Authors:** William M. Nabulyato, Hebah Alsahiem, Nigel R. Hall, Charles M. Malata

**Affiliations:** ^a^Departments of Plastic Surgery, University of Cambridge, UK; ^b^Departments of Colorectal Surgery, University of Cambridge, UK; ^c^School of Clinical Medicine, University of Cambridge, UK; ^d^Addenbrooke's Hospital Cambridge University Hospital NHS Foundtion Trust

## Abstract

**Introduction:** Heterotopic mesenteric ossification is an extremely rare condition, which often follows trauma and is frequently symptomatic. To date, there are no reports in the literature of abdominal compartment syndrome occurring after surgical resection of mesenteric calcification. The present report documents an unusual case of compartment syndrome complicating resection of extensive mesenteric calcification despite abdominal closure with the components-separation technique. **Method:** A 48-year-old man undergoing components-separation technique for posttraumatic laparostomy hernia repair (ileostomy reversal and sigmoid stricture correction) was found to have extensive heterotopic mesenteric calcification, which needed resection. **Results:** Resection of the mesenteric calcification was complicated by intraoperative hemorrhage and unplanned small bowel resection. Later the patient developed secondary hemorrhage leading to an abdominal compartment syndrome, which was successfully treated by decompression, hemostasis, and Permacol-assisted laparotomy wound closure. The patient remains symptom-free more than 2 years after surgery. **Discussion:** The case herein reported gives an account of the rare occurrence of abdominal compartment syndrome following resection of posttraumatic ectopic mesenteric ossifications. It is highly unusual in that it occurred because of “secondary hemorrhage” and despite abdominal closure with the components-separation technique, which had been undertaken precisely to prevent compartment syndrome with direct closure. It therefore highlights the need for continued clinical vigilance in complex posttraumatic cases.

Heterotopic ossification was originally described in 1692 by Guy Patin, who noted the condition in children and termed it *Myositis Ossificans Progressiva*.^1^ It was later noted in paraplegic patients injured during World War 1 by Dejerine and Ceiller^2^ in 1918, who referred to the process as *Paraosteoarthroplasty*. Although Hansen was the first to describe peritoneal ossification in 1983,^3^ the term *heterotopic mesenteric Ossification* (HMO) was coined by Wilson et al in 1999 after concluding that 5 of their postabdominal surgical cases, with small bowel obstruction associated with heterotopic bone formation in the mesentery, clinically and pathologically resembled Myositis Ossificans as previously described by Ackerman.^4^^,^^5^ Heterotopic mesenteric Ossification has since been associated with many risk factors, with severe trauma often being a reoccurring theme, see Table 1.

Intra-abdominal ossification is a rare entity with an otherwise poorly understood etiology and pathophysiology. Reported cases in literature often describe patients presenting with significant obstructive abdominal symptoms on a background of previous abdominal insult, with the process more likely to occur thereafter.^7^

We herein report how one case of HMO, in an otherwise symptomless patient requiring complex surgical intervention, was complicated further by postoperative abdominal compartment syndrome (ACS) due to secondary hemorrhage.

## CASE PRESENTATION

A 48-year-old man was admitted to hospital in mainland Europe with multiple traumatic injuries following a severe road traffic accident on the Christmas Day of 2009. He presented with a Glasgow Coma Scale of 4 to 6 with associated bilateral pneumothoraces; multiple rib fractures; left humeral, right femoral, and right patella fractures; and bilateral fractures of the L4 transverse processes and nasal bones. After initial hemodynamic stabilization, he underwent emergency cecostomy and loop ileostomy procedures for peritonitis secondary to “spontaneous” sigmoid colon perforation. The patient was subsequently transferred to the United Kingdom, where he spent 2 months on the intensive care unit (ICU) before being referred to the *plastic surgery service*. Prior to referral, he had undergone numerous exploratory laparotomies, washouts, and applications of vacuum-assisted closure (VAC) dressings. This period was further punctuated with an emergency decompression for ACS, pleural effusions, and a prolonged period of endotracheal intubation for treatment of respiratory failure.

The patient was referred to the plastic surgeons for closure of his large “iatrogenic” abdominal hernia but the size of the defect and level of exposed herniating bowel precluded direct closure, necessitating the application of a split-thickness skin graft with VAC dressing.

**Figure 1:**
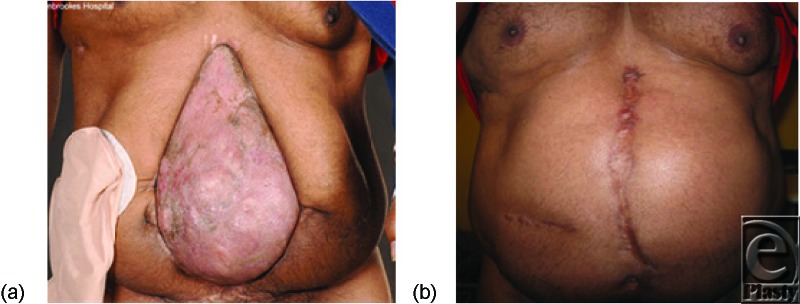
(*a*) Preoperative appearance showing ileostomy and massive central abdominal hernia with overlying mature split-thickness skin graft. (*b*) 2-year postoperative appearance following ileostomy reversal and central abdominal closure by components separation (note the improved nutritional status of the patient.)

Six months later (after maturation of the split-thickness skin graft covering the bowel; Fig 1a), the patient was admitted for formal repair of his large postlaparotomy hernia by components-separation technique^8^ with concurrent reversal of his ileostomy and resection of a posttraumatic sigmoid stricture. The patient reported no other symptoms prior to surgery.

**Figure 2:**
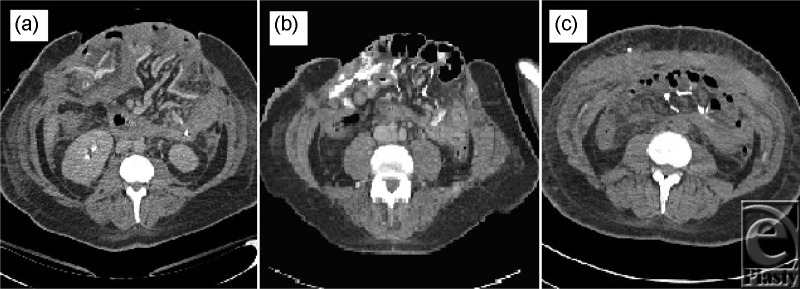
(*a*) NHS hospital admission CT abdomen, note open laparotomy wound and herniating bowel. (*b*) CT scan 6 months after initial injury shows herniated bowel, retraction of the abdominal musculature and incidental finding of diffuse peritoneal calcifications prior to components separation. (*c*) Post components separation closure CT, note remnant calcifications. CT indicates computed tomography.

Preoperative abdominal computed tomographic scans (Figs 2a and 2b) showed marked divarication (diastasis) of the anterior abdominal wall musculature and subcutaneous fat with obvious herniation of the intra-abdominal contents. No foreign bodies were detected; however, a computed tomographic scan prior to components-separation closure detected extensive peritoneal calcification not seen on previous imaging (Fig 2b).

**Figure 3:**
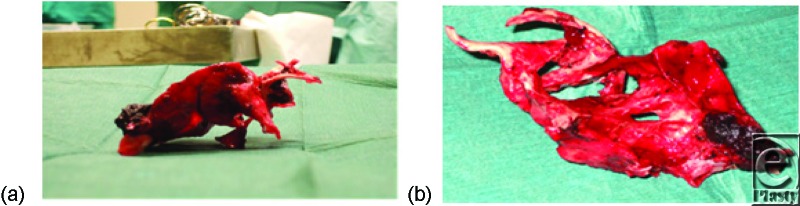
Two main pieces of ossified abdominal cavity soft tissue. One U-shaped measuring 145 × 30 mm and the other spiral shaped shown above side profile (*a*) and superior view (*b*), measuring 140 × 75 mm.

On laparotomy, some sharp, complex mesenteric peritoneal calcifications were encountered which were thought to pose too great a risk to be left in situ (Fig 3). The largest ossification was so well vascularized and intertwined with the surrounding soft tissue structures that separating it proved very difficult. Its resection coupled with the prolonged nature of the procedure led to 4 liters of blood loss and devascularization of 30 cm of small bowel segment. Following surgery, the patient was admitted to the ICU for overnight ventilation.

The patient, however, needed prolonged ventilation, and 6 days later, while on the ICU, he developed postoperative ACS from massive intraperitoneal hemorrhage requiring emergency reexplorative laparotomy and evacuation of the intraperitoneal hematoma. The abdominal wall was closed with a biological Permacol mesh and VAC dressing. He was later transferred to the ward where 2 weeks later, the abdominal skin wound was successfully closed over the underlying Permacol with no further healing problems. He was discharged home after successful postoperative rehabilitation and reports no further complications over 2 years later with the cosmetic results as seen in Fig 1B.

## DISCUSSION

Intra-abdominal ossification is a rare entity with a predilection for the mesentery that may be associated with a variety of predisposing factors of neoplastic (primary or metastatic, eg, teratomas) or nonneoplastic origin.^9^ The pathogeneses is not fully understood but literature suggests a 4-stage process for osteogenic induction.^10^ This begins with some form of primary insult and within an ideal environment leads to the inappropriate differentiation of mesenchymal cells into osteoblastic stem cells in response to still unidentified inducing agents.^11^ Trauma is a constant feature in reviewed literature, with the formation of ectopic ossification also associated with prolonged immobilization in conjunction with multiple long bone fractures and severe head injuries.^12^ Heterotropic bone formation has been reported in vertical laparotomy scars and may lead to small bowel obstruction.^13^ Differentiating the cause of postoperative mesenteric densities as shown in Table 2 is important to management and prevention of serious complications. The eleven reported cases in literature dating to 2006 shared the following common features^15^:

- All were male patients with a mean age of 56 years;
- Ten had previously undergone abdominal operations for benign conditions; and
- The same 10 also presented with small bowel obstruction secondary to mesenteric ossifications.

Interestingly, despite the increased relative risk posed and the mesenteric location of our patient's calcifications, he did not present with any obstructive signs or symptoms. The pathological findings from his excised specimens were however more in keeping with reported cases. Microscopy showed dense collections of lamellar bone, with some resembling the cortices of long bone and others resembling the medulla. These collections showed no active inflammation and were primarily within the vessels of excised fibroadipose tissue but also surrounded one normal lymph node. There were no features of malignancy in the report.

Abdominal compartment syndrome is often a fatal, multiorgan, end-stage process, resulting from poorly compensated intra-abdominal hypertension. The latter occurs when increased capillary permeability in critically ill patients with systemic inflammatory response syndrome (SIRS) leads to the sequestration of fluid into the cavity. Our patient notably survived 2 episodes of hemorrhage-induced ACS, pre and post components-separation surgery, and has been symptom-free for more than 2 years.

We concluded the innocuous appearance on radiological imaging, the significant challenge experienced during laparotomy, and the serious peri- and postoperative complications make HMO a rare but potentially fatal entity that warrants surgical intervention. Surgeons must therefore consider HMO and its complications as a differential when managing complex multitrauma patients with “suspicious” radiographic densities.

## Figures and Tables

**Table 1:** *Risk factors for heterotopic mesenteric Ossification*^6^

Risk Factor	Example
Neurological injury	To the brain and/or spinal cord
	Injection of CNS for polio and tetanus
Trauma	Operations: surgical trauma
	Fractures, Dislocations
	Burns
	Contusions
Genetic predisposition	Fibrodysplasia ossificans progressiva
	Progressive osseous heteroplasia
	Albright's hereditary osteodystrophy

**Table 2:** *Possible differential diagnoses for intra-abdominal densities seen on CT imagery after trauma or surgical exploration*^14^

Differential Diagnosis	Rational
HMO	Well defined cortices and trabecular pattern on imaging
Dystrophic calcification	Irregular, faint radiodense areas that are punctate
Osseous neoplasia	Nuclear atypia, necrosis and atypical mitotic figures
CAPD-related peritonitis	If patient on CAPD treatment
Foreign body	If postoperative
Oral contrast extravasation	Evolution on serial imagery

CAPD indicates Continuous Ambulatory Peritoneal Dialysis; CT, computed tomographic; HMO, heterotopic mesenteric Ossification.
